# Experienced stigma in Japanese outpatients with diabetes: Age and polypharmacy matter

**DOI:** 10.1097/MD.0000000000047960

**Published:** 2026-03-20

**Authors:** Haremaru Kubo, Takashi Sozu, Reina Mitsunaga, Hiromasa Hazama, Naohiro Sekikawa, Ryota Wada, Yuko Watanabe, Akira Tamura, Toshiro Yamazaki, Setsu Ohta, Susumu Suzuki, Kazuhiro Sugimoto

**Affiliations:** a Diabetes Center, Ohta Nishinouchi Hospital, Koriyama, Fukushima, Japan; b Department of Endocrinology, Diabetes and Metabolism Kitasato University School of Medicine, Sagamihara, Kanagawa, Japan; c Department of Information and Computer Technology, Faculty of Engineering, Tokyo University of Science, Katsushika-ku, Tokyo, Japan; d JURI Medical Corporation, SUGIMOTO Internal Medicine and Diabetes Clinic, Fukushima, Japan.

**Keywords:** advocacy awareness, diabetes-related stigma, experienced stigma, Japanese outpatients, polypharmacy

## Abstract

There are three types of diabetes-related stigma (DRS): perceived, experienced, and internalized, all of which negatively impact individuals with diabetes. Over the past 2 decades, research in Japan has grown, highlighting the significant clinical effects of DRS. In this study, we focused on the least-studied form, experienced stigma investigating its prevalence, clinical correlates, and the awareness of DRS and advocacy activities among Japanese people with diabetes. We conducted a single-center, cross-sectional study from April 3 to 28, 2023, at the Ohta Nishinouchi Hospital Diabetes Center in Japan, involving 114 adults with type 1 or type 2 diabetes. Participants with severe mental or physical conditions were excluded. Each participant completed a questionnaire assessing experienced stigma, the impact of diabetes on their social life, and their familiarity with the terms “diabetes stigma” and “advocacy activities.” Associations between reported stigma and demographic or clinical factors were analyzed statistically. Our findings showed that only 19.3% of participants reported a significant impact of DRS on their social life, with younger individuals and those on multiple diabetes medications more likely to report experiencing stigma. Additionally, awareness of “diabetes stigma” and “advocacy activities” was notably low among participants. In conclusion, compared to international studies, the prevalence of experienced stigma among Japanese individuals with diabetes appears lower, based on this single-center face-to-face study of outpatients. However, age and polypharmacy were identified as significant factors associated with increased reports of stigma. Despite the limitations of a single-center design, small sample size, and use of non-validated survey tools, the observed low awareness of “diabetes stigma” and “advocacy activities” underscored the need for enhanced educational initiatives by healthcare professionals and diabetes-related organizations in Japan.

## 1. Introduction

The term “stigma,” as defined by Goffman,^[1]^ refers to profoundly disqualifying attributes or conditions that deviate from social norms, resulting in social, emotional, and psychological challenges for stigmatized individuals or groups. People living with diabetes may face stigma, often perceiving or experiencing embarrassing, shameful, and depressive episodes in various social contexts, such as social gatherings, job interviews or promotions, marital engagements, pregnancies, health policies/legislations, or healthcare settings. Until at least until 2013,^[2]^ research on diabetes-related stigma (DRS) was limited worldwide. However, DRS is now recognized as a significant clinical challenge, with efforts focused on overcoming widespread misconceptions about insulin treatment and diabetes itself.^[3]^

DRS can be classified into three types: perceived (social, public, or felt stigma), experienced (self-perceived or enacted stigma), and self-stigma (internalized stigma).^[4,5]^

1. **Perceived stigma** involves anticipating social judgment, adverse reactions, or discrimination that may not actually occur but is expected by people with type 1 diabetes (T1D),^[6]^ though it has been shown to significantly reduce self-efficacy in those with type 2 diabetes (T2D).^[7]^ Social stigma has also been associated with a higher body mass index (BMI) (weight in kilograms divided by height in meters squared), elevated hemoglobin A1c (HbA1c), and poorer self-reported blood glucose control in both T1D and T2D individuals.^[8]^
2. **Experienced stigma** refers to the actual episodes of social discrimination and participation restrictions due to a lack of societal acceptance of diabetes.^[9]^ Experienced stigma has been associated with younger age, higher levels of psychological distress, more pronounced depressive symptoms, and lower levels of social support in individuals with T1D and T2D.^[9]^
3. **Self-stigma** reflects the internalization of a stigmatized identity and the acceptance of these views as part of one self-perception.^[5]^ Self-stigma has been associated with depressive and anxiety symptoms and diabetes-related distress in T1D and T2D individuals,^[10]^ quality of life in those with T2D,^[11]^ and reduced social support in people with T2D.^[12]^

In Japan, research on DRS has increased significantly over the past two decades. In 2003, Sato et al published a qualitative study that first explored the socio-psychological impacts of DRS.^[13]^ Subsequently, Kato et al developed the Japanese version of the self-stigma scale, identifying associations between self-stigma and reduced self-esteem, lower self-efficacy, and increased depressive symptoms in people with T2D.^[14]^ They also reported correlations between self-stigma and higher HbA1c levels, decreased self-care behaviors,^[5]^ reduced health-related quality of life,^[15]^ diminished self-care activity,^[16]^ and a disease duration of 11 to 15 years.^[17]^ Notably, increased psychosocial complications in youth, adolescents, and young adults with T2D have been widely documented internationally.^[18]^ Additionally, the patients who undergoing polypharmacy may be more likely to recognize self-stigma.^[19]^ These factors could represent important considerations in the management of individuals with T2D. However, despite these insights, information regarding the prevalence and clinical correlates of experienced stigma among Japanese people with diabetes remains limited. Furthermore, the questionaries used in the previous studies, such as the Japanese version of the self-stigma scale, include a large number of items, (e.g., 39 questions),^[14]^ which may pose practical challenges for routine clinical use. Therefore, there is a growing need for simpler and more feasible assessment tools.

A framework for diabetes advocacy activities has been proposed to identify challenges and gaps in current diabetes advocacy, including efforts to reduce pervasive stigma and misconceptions surrounding diabetes.^[20]^ Since August 4, 2019, the Japan Diabetes Society (JDS) and Japan Association for Diabetes Education and Care (JADEC) have formed a joint committee to address and overcome DRS. Advocacy efforts are considered to play an important role in improving healthcare outcomes in other conditions, such as infectious diseases and cancers.^[20]^ However, even among general physicians, awareness of the terms “stigma” and “advocacy” was reported to be limited to approximately 40% in 2022.^[21]^ It is therefore plausible that awareness of these concepts is considerably lower among the general public. Moreover, no studies have examined the awareness of these concepts among Japanese people with diabetes.

This single-center questionnaire study aimed to explore the prevalence and clinical correlates of experienced stigma assess the awareness of DRS and advocacy activities among Japanese individuals with diabetes.

## 2. Material and methods

### 2.1. Ethics approval

This study was conducted with the approval of the Ethics Committee of Ohta Nishinouchi Hospital (approval no. 25; approval date, January 11, 2023), followed the Declaration of Helsinki (1964) and its later amendments, as well as the ethical guidelines for medical and health research involving human subjects issued by the Ministry of Health, Labor and Welfare of Japan in 2017. The study registry details are as follows: study registration number UMIN000052489.

### 2.2. Study period and location

A single-center, cross-sectional study was conducted in April 2023 at the Ohta Nishinouchi Hospital, located in Koriyama, the second most populous city in Japan northeast (Tohoku) district.

### 2.3. Participants and procedure

One hundred fourteen eligible Japanese adults (age 18 years or older) with T1D or T2D were recruited from the outpatient clinic of the diabetes center at Ohta Nishinouchi Hospital. The sample size was determined based on research feasibility, and a minimum of 100 participants was targeted considering the burden on physicians, outpatient staff, and the process of obtaining informed consent. Exclusion criteria, determined by the attending physicians (HK, SO, and KS), included mental health or psychological disorders, conditions requiring emergency treatment (e.g., acute infection or trauma), malignant diseases, or unwillingness to participate. Before enrollment, the study overview was explained to participants, and written informed consent for this study was obtained.

### 2.4. Questionnaire contents

To assess self-stigma and other categories of diabetes-related stigma (DRS) among people with diabetes in Japan, we used 2 established questionnaires. However, since no validated questionnaire existed to specifically measure experienced stigma or awareness of advocacy initiatives by the JDS and JADEC, we designed an unvalidated, four-question survey as follows:

Q1: Assessing the impact of diabetes on social life: “Do you feel that diabetes has hindered your active social life?”

Q2: Perception or experience of DRS: “Do you feel you have faced unjustified discrimination or prejudice from others due to diabetes?”

Q3: Awareness of “diabetes stigma” promoted by the JDS and JADEC:: “How familiar are you with the ‘diabetes stigma’ awareness efforts by the JDS and JADEC?”

Q4: Awareness of “advocacy activities” by the JDS and JADEC: “How familiar are you with the ‘advocacy activities’ by the JDS and JADEC?”

All questions were administered in Japanese, with one physician (HH) sitting beside the participant to clarify any misunderstandings. Responses to Q1 and Q2 were rated on a 5-point Likert scale of agreement (1 = strongly agree to 5 = strongly disagree), while Q3 and Q4 used a 4-point scale (1 = very well to 4 = not well at all).

### 2.5. Clinical characteristics

Medical records for 114 participants were reviewed to obtain information on age, sex, height, weight, diabetes type, known diabetes duration, HbA1c, and diabetes medications (oral hypoglycemic agents, glucagon-like peptide-1 receptor agonists (GLP-1RAs, either oral or injectable), and insulin) on the date of questionnaire completion.

### 2.6. Statistical analysis

Participants’ characteristics were summarized as the mean and SD or the median [interquartile range] for continuous variables and the numbers (percentages) for categorical variables. Associations between age, sex, BMI, diabetes type, diabetes duration, HbA1c, and antidiabetic medications (insulin/GLP-1RA/other OHA) with the Likert scale responses were analyzed using the Mantel test, generalized Mantel, linear trend test, or Fisher exact test, as appropriate and summarized using Cramer V. As this study was designed to generate new hypotheses rather than to validate predefined ones, rigorous adjustment for multiple comparisons was not performed. Instead, we interpreted the *P*-values quantitatively, taking into account the total number of hypothesis tests, and focused on the clinical parameters showing significance at *P*-value < .05. Two biostatisticians (TS and RM) conducted all the statistical analyses using R software (version 4.3.3).

## 3. Results

### 3.1. Participant demographics and clinical parameters

The study included 114 participants, comprising 77 males (67.5%) and 37 females (32.5%), with a mean (SD) age of 61 (15) years and BMI of 24.5 (4.6) kg/m^2^ (Table 1). Among them, 21 participants (18.4%) had type 1 diabetes (T1D), and 93 (81.6%) had type 2 diabetes (T2D). The median (min, max) duration of diabetes was 10.0 [0.5, 40.0] years, mean (SD) HbA1c was 7.3 (0.8)%, and 39 participants (34.2%) were receiving insulin therapy. There were 30 missing responses for “Diabetes duration” in the baseline characteristics. As these missing values are assumed to be missing completely at random, the potential impact of this bias is considered minimal. No other missing data were identified in the study.

**Table 1:** Characteristics of the study participants.

*Clinical characteristics*	
Age (years)	61 ± 15
Sex (male/female)	77/37 (67.5/32.5)
BMI (kg/m^2^)	24.5 ± 4.6
Type of diabetes (type 1/type 2)	21 (18.4)/93 (81.6)
Diabetes duration (years)	10.0 [0.5, 40.0]
HbA1c (%)	7.3 ± 0.8
*Diabetes medications*	
Insulin	39 (34.2)
Oral GLP-1RA	12 (10.5)
Injectable GLP-1RA	17 (14.9)
Other OHA	91 (79.8)

Data are number (%), mean ± SD, or median (min, max).

Antidiabetic agents include metformin, pioglitazone, α-glucosidase inhibitor, dipeptidyl-peptidase 4 inhibitor, sodium-glucose transporter 2 inhibitor, imeglimin, glinide, and glimepiride.

BMI = body mass index, GLP-1RA = glucagon-like peptide 1 receptor agonist, HbA1c = hemoglobin A1c, OHA = oral hypoglycemic agent.

### 3.2. The prevalence of experienced stigma

**Figure 1.**
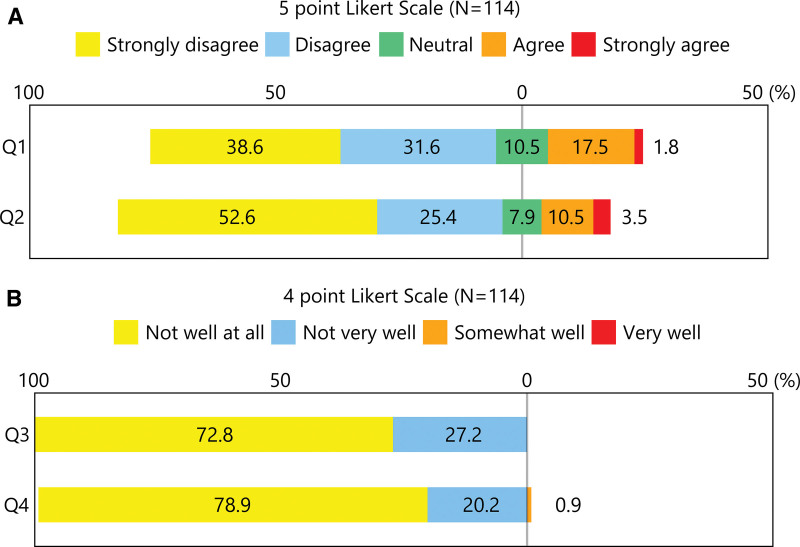
Horizontal diverging stacked bar charts of Likert scale responses.

Figure 1A shows the participants’ responses to Q1 and Q2 on a 5-point Likert scale. Only 2 participants (1.8%) and 4 participants (3.5%) responded with “strongly agree” to Q1 and Q2, respectively, while 20 participants (17.5%) and 12 participants (10.5%) responded with “agree.” The majority (70.2% and 78.0%) responded with either “strongly disagree” or “disagree” to Q1 and Q2, respectively.

This figure presents the Likert scale responses as percentages for each question.

(A) Responses to Q1 and Q2:

Q1: Assessing the impact of diabetes on social life — “Do you feel that diabetes has hindered your active social life?”

Q2: Perception or experience of DRS — “Do you feel you have faced unjustified discrimination or prejudice from others due to diabetes?”

(B) Responses to Q3 and Q4:

Q3: Awareness of “diabetes stigma” promoted by the JDS and JADEC — “How familiar are you with the ‘diabetes stigma’ awareness efforts by the JDS and JADEC?”

Q4: Awareness of “advocacy activities” by the JDS and JADEC — “How familiar are you with the ‘advocacy activities’ by the JDS and JADEC?”

Abbreviations: DRS, diabetes-related stigma; JDS, Japan Diabetes Society; JADEC, Japan Association for Diabetes Education and Care.

Among the 16 participants who responded with either “strongly agree” or “agree” to Q2, they chose the situations in which they reported experiencing discrimination or prejudice included employment (*n* = 10), dining events (*n* = 6), job promotion (*n* = 5), health insurance enrollment (*n* = 5), others (*n* = 4), marriage (*n* = 2), loan application (*n* = 2), and hospital (*n* = 1) (Fig. S1, Supplemental Digital Content, https://links.lww.com/MD/R521).

### 3.3. Awareness of DRS and advocacy activities

Figure 1B displays the participants’ responses to Q3 and Q4 on a 4-point Likert scale. None of the participants responded “very well” to Q4, and only one responded “somewhat well.” Nearly, all participants (100% for Q3 and 99.1% for Q4) responded with either “not well at all” or “not very well,” indicating limited awareness of DRS and advocacy activities by JDS and JADEC.

### 3.4. Associations of clinical characteristics with experienced stigma

Due to the low percentages of participants responding with “strongly agree” to Q1 and Q2, which were very small (1.8% and 3.5%, respectively), we combined the responses of “strongly agree” and “agree” for further analyses. Additionally, one participant who responded “somewhat well” to Q4 was excluded, limiting analyses with either “not well at all” or “not very well” to Q3 and Q4.

**Figure 2.**
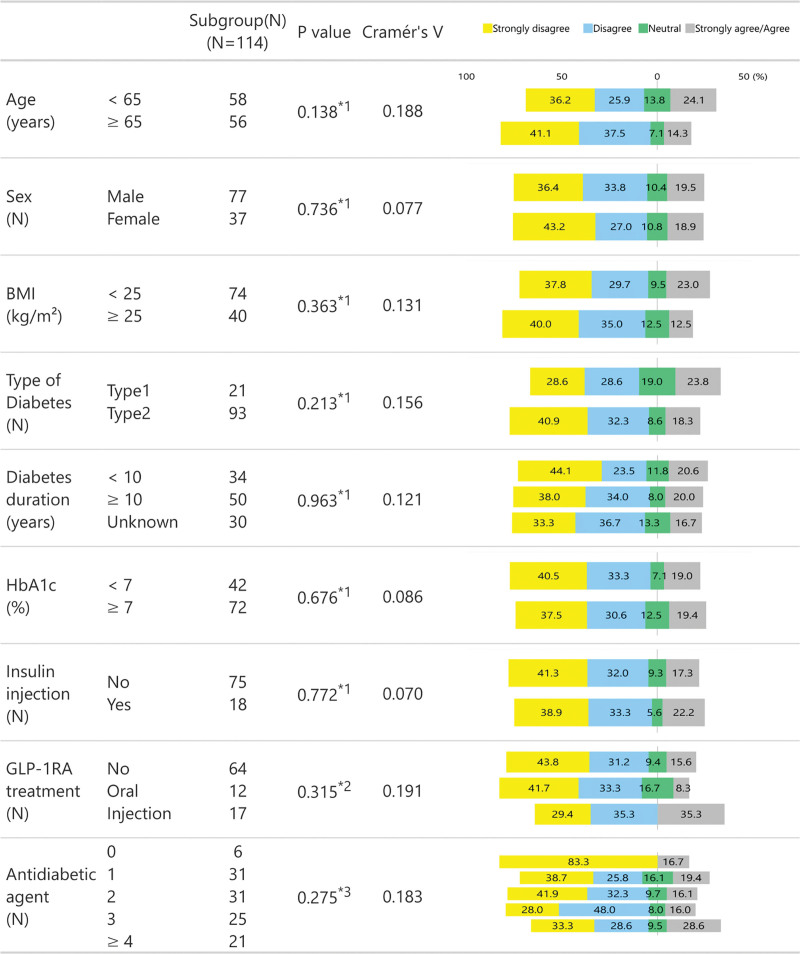
Likert scale responses to Q1 across stratified subgroups.

**Figure 3.**
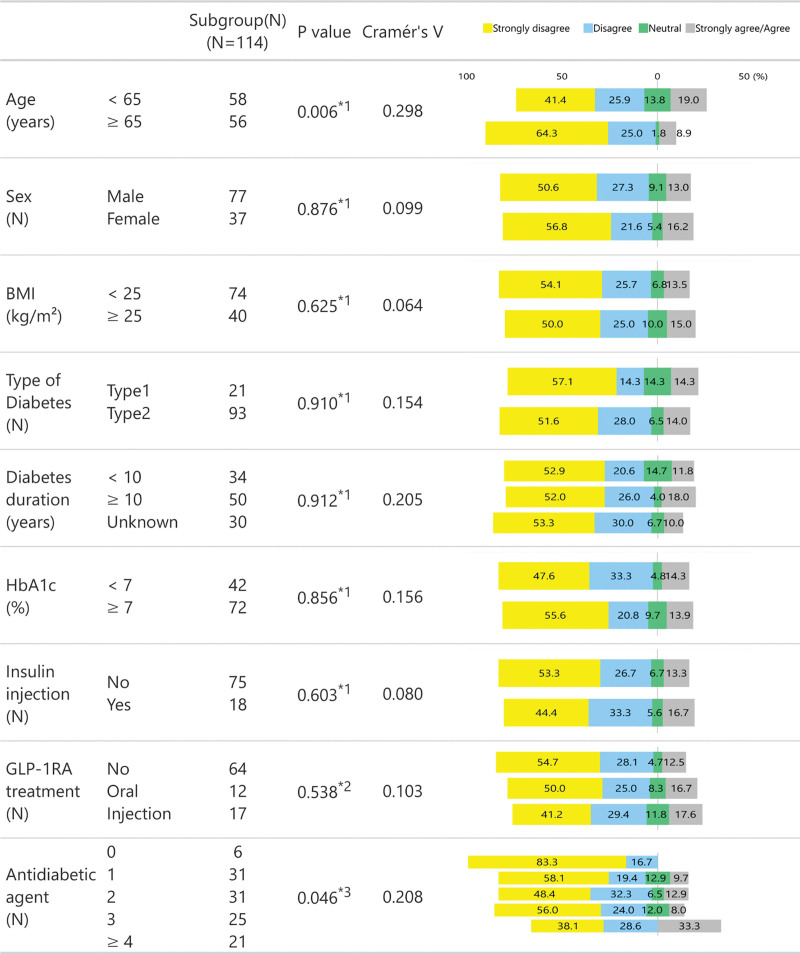
Likert Scale Responses to Q2 Across Stratified Subgroups.

**Figure 4.**
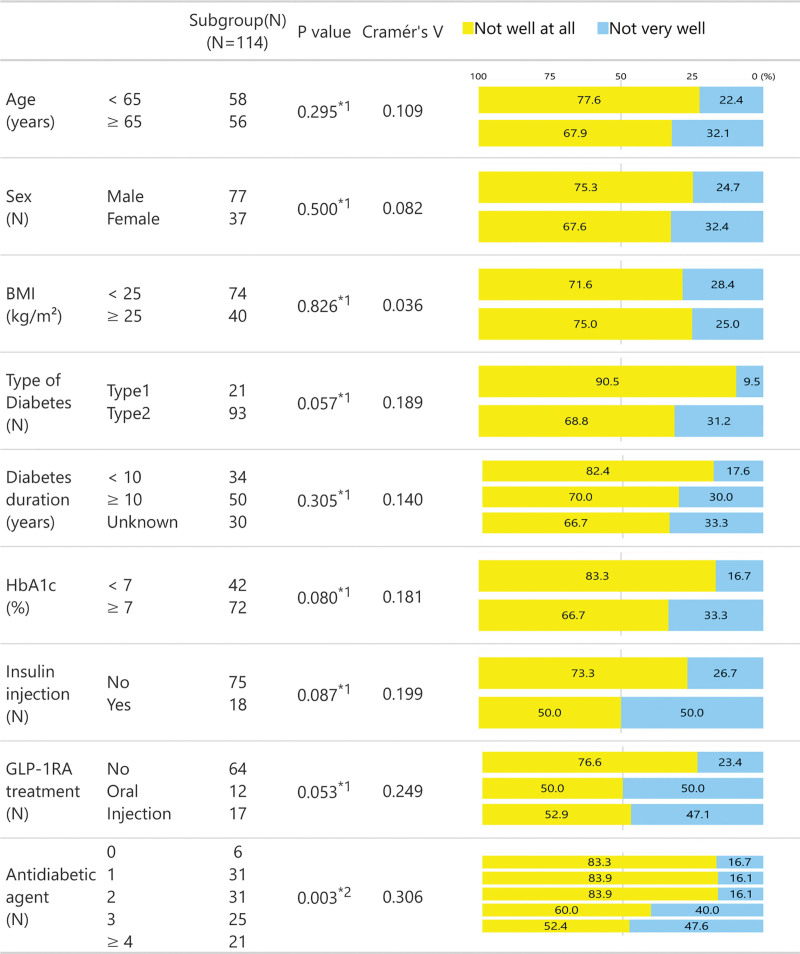
Likert scale responses to Q3 across stratified subgroups.

**Figure 5.**
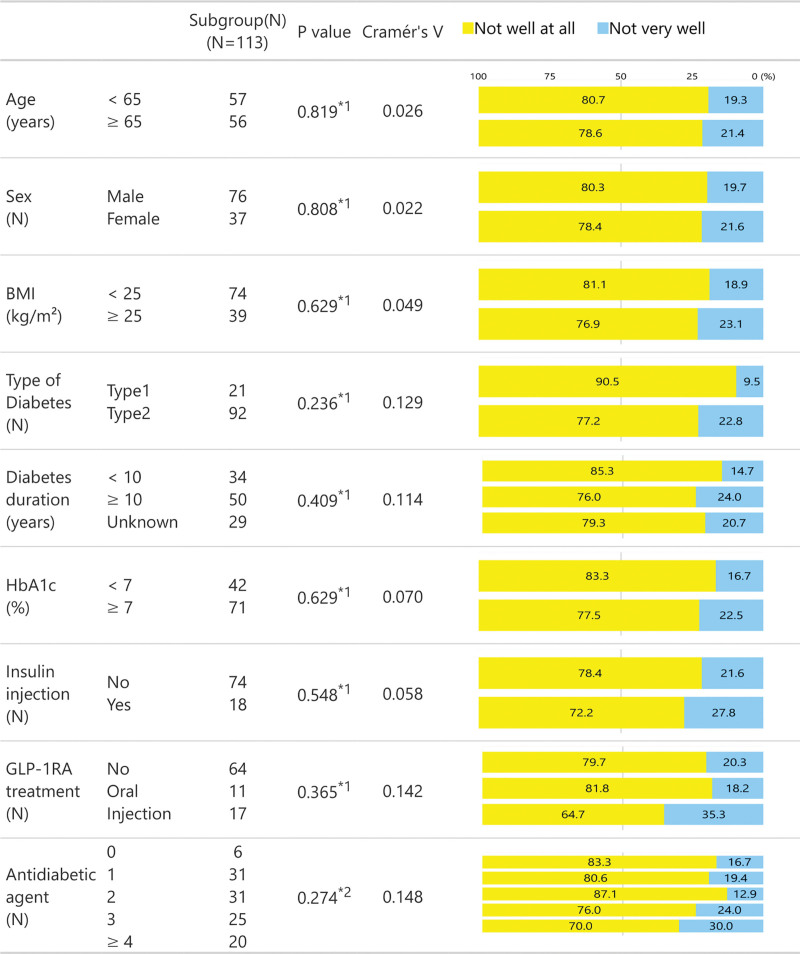
Likert scale responses to Q4 across stratified subgroups.

We examined differences in Likert scale scores for Q1–Q4 (Figs. 2–5) between subgroups stratified by age (<65 vs ≥65 years of age), sex, BMI (<25 s, ≥25 kg/m^2^), diabetes type (T1D vs T2D), diabetes duration (<10 vs ≥10 years), HbA1c (<7 vs ≥7%), insulin use (no vs yes), GLP-1RA treatment (none, oral or injection), and the number of antidiabetic agents. For insulin and GLP-1RA stratifications, T1D participants (*n* = 21) were excluded as all were treated with insulin injections and without GLP-1RAs.

This figure compares responses to Q1 across various stratified subgroups using horizontal diverging stacked bar charts.

^*1^P-values are calculated using the Mantel test, ^*2^generalized Mantel test, and ^*3^linear trend test.

Participants with unknown diabetes duration were excluded from analyses involving diabetes duration stratification.

BMI = body mass index; GLP-1RA = glucagon-like peptide 1 receptor agonist; HbA1c = hemoglobin A1c.

This figure compares responses to Q2 across various stratified subgroups using horizontal diverging stacked bar charts.

^*1^*P*-values are calculated using the Mantel test, ^*2^generalized Mantel test, and ^*3^linear trend test.

Participants with unknown diabetes duration were excluded from analyses involving diabetes duration stratification.

BMI = body mass index; GLP-1RA = glucagon-like peptide 1 receptor agonist; HbA1c = hemoglobin A1c.

This figure compares responses to Q3 across various stratified subgroups using horizontal diverging stacked bar charts.

^*1^*P*-values are calculated using Fisher exact test or ^*2^linear trend test.

The participants with unknown diabetes duration were excluded from analyses involving diabetes duration stratification.

BMI = body mass index; GLP-1RA = glucagon-like peptide 1 receptor agonist; HbA1c = hemoglobin A1c.

This figure compares responses to Q4 across various stratified subgroups using horizontal diverging stacked bar charts.

^*1^*P*-values are calculated using Fisher exact test or ^*2^linear trend test.

The participants with unknown diabetes duration were excluded from analyses involving diabetes duration stratification.

BMI = body mass index; GLP-1RA = glucagon-like peptide 1 receptor agonist; HbA1c = hemoglobin A1c.

There were no significant differences in Likert scores for Q1 across any stratified subgroups, as reflected in the Cramér *V* values (all <0.2), suggesting negligible associations (Fig. 2). However, for Q2, significant differences were observed between the participants aged <65 years and ≥65 years (*P* = .006) and between groups using varying numbers of antidiabetic agents (*P* = .046). Participants aged <65 years and those using ≥4 antidiabetic agents were more likely to respond with either “strongly agree” or “agree” to Q2 than those aged ≥65 years (19.0% vs 8.9%) and those using fewer antidiabetic agents (33.3% vs 0–12.9%), respectively. However, all Cramér *V* values were below 0.3, indicating minimal to weak associations across all comparisons (Fig. 3).

Further analysis revealed that 40.0% of the participants aged <65 years and using ≥4 antidiabetic agents responded with either “strongly agree” or “agree” to Q2 compared to only 4.4% of those aged ≥65 years and using <4 agents (*P* = .006). Such an additive effect of younger age and high medication burden on experiencing DRS is shown in Fig. S2, Supplemental Digital Content, https://links.lww.com/MD/R521.

For Q3, significant differences emerged in the number of antidiabetic agents. Participants using ≥3 antidiabetic agents were more likely to respond with “not very well” to Q3 compared to those on fewer agents (40.0% and 47.6% vs 16.1–16.7%, respectively). Participants on insulin injections (50.0% vs 26.7%) or oral and injectable GLP-1RA treatments (50.0% and 47.1% vs 23.4%, respectively) also tended to respond with “not very well” to Q3. Notably, Cramér *V* values for GLP-1RA treatment (0.249) and the number of antidiabetic agents (0.306) suggest weak but potentially meaningful associations (Fig. 4). No significant subgroup differences were observed for Q4, consistent with Cramér *V* values, all of which were below 0.2 (Fig. 5).

## 4. Discussion

In this single-center, cross-sectional study, we surveyed 114 diabetes outpatients to assess the frequency and settings in which they experience stigma and their familiarity with DRS and advocacy efforts by the JDS and JADEC. Only 19.3% of participants agreed with the statement, “Do you feel that diabetes has hindered your active social life?” and 14.0% agreed with the question, “Do you feel you have faced unjustified discrimination or prejudice from others due to diabetes?.” Furthermore, none of the participants were well acquainted with the “diabetes stigma” promoted by the JDS and the JADEC, and only one participant was somewhat familiar with their “advocacy activities.”

In a comparable study from Switzerland involving 3347 people with diabetes, 68.5% reported experiencing discrimination due to their condition.^[9]^ In the United States, a large-scale online survey among 12,000 people with diabetes found that 76.0% with T1D and 52.0% with T2D experienced stigma.^[8]^ Another Canadian online study reported that 65.5% of 380 adolescents and young adults with T1D felt stigmatized.^[22]^ In contrast, a Japanese cross-sectional online survey of 510 T2D participants revealed that 32.9% had experienced diabetes-related shame, a key aspect of stigma.^[23]^ In the present face-to-face survey conducted among outpatients with diabetes, the reported prevalence of DRS was even lower than in these international and online studies. This discrepancy may partly reflect cultural and methodological differences. In Japanese clinical settings, individuals often internalize experiences of discrimination and prejudice, viewing them as personal issues to be managed independently.^[24]^ Such tendencies may lead to underreporting, especially in face-to-face interviews, where disclosing emotional burdens to others is culturally restrained. These factors may contribute to the observed lower awareness of stigma in this study. Further research is warranted to clarify whether the differences in DRS prevalence reflect true cultural distinctions, methodological effects, or differences in participant background.

Our findings also indicated that DRS was most reported in the workplace. A previous study involving 705 workers with T2D found that 23% had chosen not to disclose their diabetes to their employer.^[25]^ Non-disclosure was associated with more sickness absence, longer disease duration, greater use of antidiabetic medications, higher educational levels, and a perception of not being respected by superiors. These findings underscore the importance of addressing workplace environments to mitigate unequal treatment and opportunities for people with diabetes.

In our survey, younger participants (<65 years) and those using ≥4 antidiabetic agents were more likely to report experiencing unjustified discrimination or prejudice. These factors appeared to have an additive effect on the likelihood of experiencing DRS (Fig. S2, Supplemental Digital Content, https://links.lww.com/MD/R521). Previous studies have also identified younger age as a risk factor for both social and self-stigma in diabetes.^[9,17]^ A recent meta-analysis suggested that younger individuals may be particularly vulnerable to psychological distress, as they are often primary earners and may face social and work-related restrictions due to their condition.^[26]^ Previous large-scale studies have also linked polypharmacy with psychological distress,^[27,28]^ greater disease severity, and macrovascular complications.^[29]^ While direct evidence is limited, it is reasonable to hypothesize that these polypharmacy-related challenges—such as complex treatment regimens and increased self-management burden—may contribute to heightened perceptions of stigma among patients. One hospital-based study reported that individuals with higher T2D-related distress were less likely to adhere to their medication regimens.^[27,28]^ In our study, a potential association was observed between polypharmacy and awareness of DRS (Fig. 4), suggesting that clinicians should remain mindful of the psychosocial implications of complex medication regimens when caring for individuals with diabetes. Future research is needed to more clearly define the relationship between polypharmacy and DRS.

Insulin therapy, known for its psychological and treatment burdens,^[30]^ is often associated with DRS.^[31,32]^ However, our findings did not support this association. This discrepancy might stem from differences in our sample characteristics, such as a relatively lower proportion of insulin users (36.0%) and a lower mean HbA1c (7.3%) compared to previous studies (where insulin use was 37.8%–50.0% and HbA1c ranged from 8.0% to 8.7%–8.9%).^[31,32]^ It should be noted, however, that this study represents a preliminary analysis, and several of the findings remains speculative. Moreover, the methodological robustness of the present data has not yet been fully established.

This study is also the first to assess the awareness of the terms “diabetes stigma” and “advocacy activities” among people with diabetes in Japan, and our findings indicate that awareness is notably low. These results suggest that there may be room for improvement in the initiatives related to stigma and advocacy efforts initiated by JDS and JADEC. Future studies should also confirm the external validity of our findings across different areas (e.g., rural vs urban) and healthcare settings (e.g., primary vs specialized facilities).

This study has several limitations.

1. The limited sample size from a single-center study may reduce statistical power and introduce sampling bias.
2. The survey included a relatively small number of items, which may not fully capture the psychological and social burdens experienced by participants, potentially resulting in measurement bias.
3. Key clinical and socioeconomic variables, such as diabetes-related complications, education, income, occupation, and marital status, were not collected and may act as unmeasured confounding factors.
4. The exploratory nature of the analysis and lack of adjustments for multiple comparisons raise the potential for type I error.
5. Finally, the methodological validity of the instruments used requires further investigation, and the clinical implications of the current findings remain somewhat speculative.

## 5. Conclusions

This face-to-face survey, consisting of 4 questions, represents the first report to explore experienced stigma among Japanese diabetes outpatients at a single center in Koriyama City. The findings suggest a lower prevalence of DRS compared to prior international studies, with the workplace identified as the most common setting where stigma was encountered. Despite the use of an unvalidated tool, younger age and polypharmacy were significantly associated with experiences of DRS, suggesting the need for targeted interventions addressing these at-risk groups. Additionally, low awareness of terms such as “diabetes stigma” and “advocacy” highlights the importance of enhanced educational outreach by healthcare professionals and diabetes-related organizations in Japan. Further studies involving larger, multicenter cohorts are necessary to validate the current findings and assess the reliability and utility of the survey tool used in this study.

## Acknowledgments

The authors thank all the study participants for attending this study. The authors gratefully acknowledge Izumi Furukawa’s contribution to data collection and analysis.

## Author contributions

**Data curation:** Haremaru Kubo, Hiromasa Hazama, Naohiro Sekikawa, Ryota Wada, Yuko Watanabe, Akira Tamura, Toshiro Yamazaki, Setsu Ohta, Susumu Suzuki, Kazuhiro Sugimoto.

**Conceptualization:** Kazuhiro Sugimoto.

**Funding acquisition:** Kazuhiro Sugimoto.

**Formal analysis:** Haremaru Kubo, Takashi Sozu, Reina Mitsunaga, Hiromasa Hazama.

**Investigation:** Haremaru Kubo, Hiromasa Hazama, Kazuhiro Sugimoto.

**Methodology:** Takashi Sozu, Kazuhiro Sugimoto.

**Project administration:** Kazuhiro Sugimoto.

**Resources:** Kazuhiro Sugimoto.

**Software:** Reina Mitsunaga.

**Supervision:** Takashi Sozu, Kazuhiro Sugimoto.

**Validation:** Kazuhiro Sugimoto.

**Writing – original draft:** Haremaru Kubo, Takashi Sozu, Hiromasa Hazama.

**Writing – review & editing:** Takashi Sozu, Hiromasa Hazama, Kazuhiro Sugimoto.

## Supplementary Material


