# Supplementary material for: Experienced stigma in Japanese outpatients with diabetes: Age and polypharmacy matter
Source: Medicine (Baltimore). 2026 Mar 20;105(12):e47960. doi: 10.1097/MD.0000000000047960 (PMC13008152; doi:10.1097/MD.0000000000047960)
Supplement: Supplementary file 1 [file medi-105-e47960-s001.docx]

**Title: Experienced stigma in Japanese outpatients with diabetes: Age and polypharmacy matter**

**Short title: Experienced stigma in diabetes**

Haremaru Kubo MD, PhD ^1, 3^, Takashi Sozu PhD ^2^, Reina Mitsunaga ^2^, Hiromasa Hazama MD ^1^, Naohiro Sekikawa MD ^1^, Ryota Wada MD ^1^, Yuko Watanabe MD ^1^, Akira Tamura MD, PhD ^1^, Toshiro Yamazaki MD ^1^, Setsu Ohta MD, PhD ^1^, Susumu Suzuki MD, PhD^1^, Kazuhiro Sugimoto MD, PhD ^1*^

^1^ Diabetes Center, Ohta Nishinouchi Hospital, Koriyama, Fukushima, Japan

^2^ Department of Information and Computer Technology, Faculty of Engineering, Tokyo University of Science, Katsushika-ku, Tokyo, Japan

^3^ Department of Endocrinology, Diabetes and Metabolism Kitasato University School of Medicine, Sagamihara, Kanagawa, Japan

*Corresponding author: Kazuhiro Sugimoto MD, PhD

E-mail: kasugi@crux.ocn.ne.jp

*Supplemental Content*

**Material and Methods**

If the participants responded with "agree" to 2^nd^ question (Q2), they were asked to specify social situations where discrimination or prejudice occurred, including dining events, employment, job promotions, marriage, loan applications, health insurance enrollment, hospitals, or other settings.

Participants' characteristics were classified into the binary variables as Age < 65 or ≧ 65 years-old and using antidiabetic agents < 4 or ≧ 4 numbers with the Likert scale responses. These were analyzed using Fisher's exact test.

**Figure S1. Situations where discrimination or prejudice occurs**

**
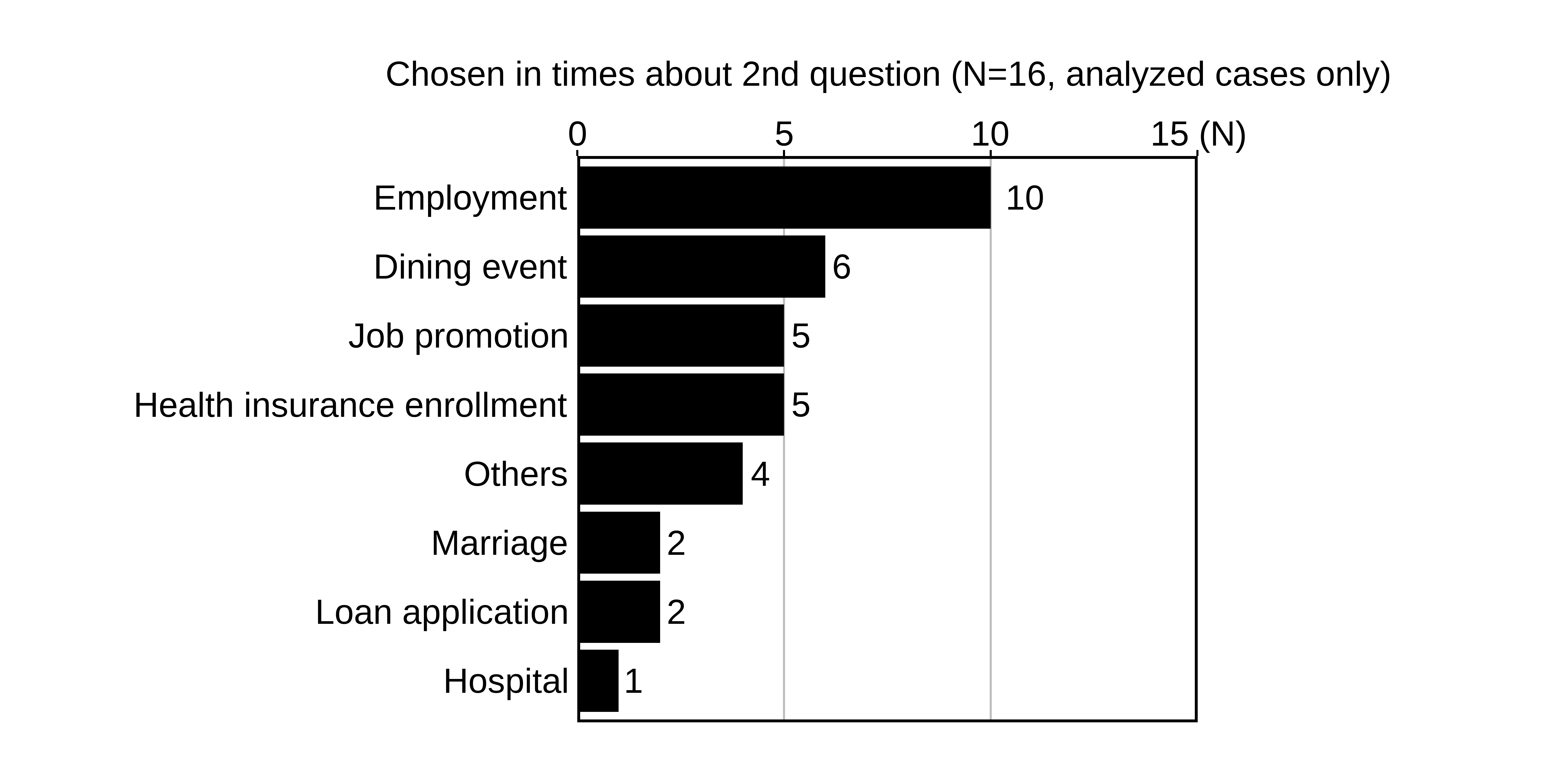
**

**Figure S2. Association between perception or experience of DRS and age or polypharmacy.**

**
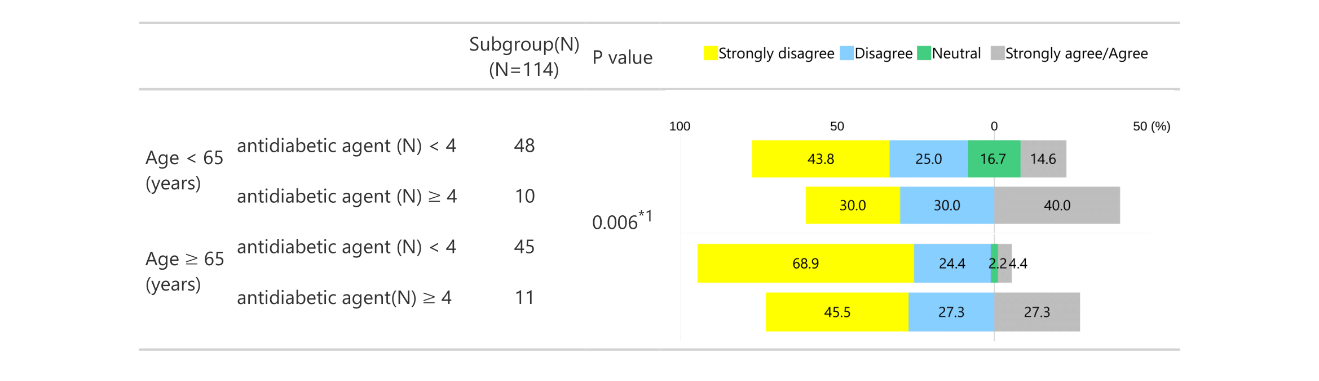
**
